# Potential Dual Role of West Nile Virus NS2B in Orchestrating NS3 Enzymatic Activity in Viral Replication

**DOI:** 10.3390/v13020216

**Published:** 2021-01-31

**Authors:** Alanna C. Tseng, Vivek R. Nerurkar, Kabi R. Neupane, Helmut Kae, Pakieli H. Kaufusi

**Affiliations:** 1 Department of Tropical Medicine, Medical Microbiology and Pharmacology, John A. Burns School of Medicine, University of Hawaii at Manoa, Honolulu, HI 96813, USA; acytseng@hawaii.edu; 2 Molecular Biosciences and Bioengineering Graduate Program, College of Tropical Agriculture and Human Resources, University of Hawaii at Manoa, Honolulu, HI 96822, USA; 3 Pacific Center for Emerging Infectious Diseases Research, John A. Burns School of Medicine, University of Hawaii at Manoa, Honolulu, HI 96813, USA; 4 Division of Math and Sciences, Leeward Community College, University of Hawaii, Pearl City, HI 96782, USA; kabi@hawaii.edu (K.R.N.); helmut@hawaii.edu (H.K.)

**Keywords:** flavivirus, West Nile Virus, membrane structures, endoplasmic reticulum, NS3, NS2B

## Abstract

West Nile virus (WNV) nonstructural protein 3 (NS3) harbors the viral triphosphatase and helicase for viral RNA synthesis and, together with NS2B, constitutes the protease responsible for polyprotein processing. NS3 is a soluble protein, but it is localized to specialized compartments at the rough endoplasmic reticulum (RER), where its enzymatic functions are essential for virus replication. However, the mechanistic details behind the recruitment of NS3 from the cytoplasm to the RER have not yet been fully elucidated. In this study, we employed immunofluorescence and biochemical assays to demonstrate that NS3, when expressed individually and when cleaved from the viral polyprotein, is localized exclusively to the cytoplasm. Furthermore, NS3 appeared to be peripherally recruited to the RER and proteolytically active when NS2B was provided in trans. Thus, we provide evidence for a potential additional role for NS2B in not only serving as the cofactor for the NS3 protease, but also in recruiting NS3 from the cytoplasm to the RER for proper enzymatic activity. Results from our study suggest that targeting the interaction between NS2B and NS3 in disrupting the NS3 ER localization may be an attractive avenue for antiviral drug discovery.

## 1. Introduction

West Nile virus (WNV), a member of the *Flavivirus* genus, is a mosquito-borne virus that can cause meningitis, encephalitis, and acute flaccid paralysis, resulting in long-term neurological damage [1,2]. Unfortunately, treatment is limited to supportive care, which necessitates the development of vaccines and effective antivirals to combat WNV infection. 

WNV is an enveloped virus consisting of a single-stranded, positive-sense RNA genome, which is directly translated into three structural proteins (capsid, precursor membrane and envelope) and seven nonstructural (NS) proteins (NS1, NS2A, NS2B, NS3, NS4A, NS4B and NS5) [3,4]. The structural proteins are involved in the entry, assembly, and maturation of the virus particle [5,6,7]. The NS proteins comprise the virus replication complex localized in virus-induced compartments at the rough endoplasmic reticulum (ER) [8,9,10]. Following translation of the polyprotein, NS1 translocates into the ER lumen and acts as a cofactor for viral RNA replication [8,11,12]. NS2A, NS2B, NS4A, and NS4B have no clear roles, but each contain at least two membrane-spanning regions that may facilitate the assembly and/or anchorage of viral replication complexes to the rough ER [13,14,15,16]. In contrast, NS3 and NS5 are soluble NS proteins with known enzymatic roles required for virus replication [17,18,19,20].

The NS3 protein consists of a distinct protease domain for polyprotein processing, and helicase and triphosphatase domains for viral RNA synthesis [18]. The protease domain is at the N-terminal end, while the helicase, nucleoside triphosphatase (NTPase) and RNA triphosphatase (RTPase) domains are at the C-terminal end of NS3 [18,21]. The NS3 protease is only active when it is tethered to its cofactor, NS2B [22]. This NS2B/NS3 complex is essential for the cleavage at the NS2A–NS2B, NS2B–NS3, NS3–NS4A and NS4B–NS5 junctions [23], and for the selective unwinding of replicating viral RNA [8,24]. However, due to its lack of transmembrane domains, once NS3 is cleaved from the polyprotein, it is either released into the cytoplasm [25] or retained at the ER where its enzymatic domains are critically needed [26,27]. It is known that NS2B is a cofactor for NS3 proteolytic function but its potential role in recruiting and/or stabilizing NS3 in the ER has not been fully explored. 

WNV has four transmembrane proteins, NS2A, NS2B, NS4A and NS4B, that could potentially recruit NS3 to the ER [13,16,28,29], but only NS2B, NS4A and NS4B have some degree of interaction with NS3 in the presence of the virus-induced compartments [30,31,32]. The interaction of NS4A and NS4B with NS3 may be facilitated by the initiation of the membrane compartments during infection, which is supported by a study using plasmid constructs showing that NS4A and NS4B do not interact with NS3 [33]. Published data have shown that NS2B has exposed hydrophilic region(s) at the cytoplasmic face of the ER that interact with NS3 in the absence of the virus-induced compartments [34]. NS2B is an essential cofactor for NS3 protease, and in the absence of NS2B, the NS3 protease domain is inactive [22,34,35,36]. Based on the strict interaction of NS3 and NS2B, we hypothesize that NS2B not only serves as a critical cofactor for viral protease activity, but also as a viral factor that may recruit NS3 from the cytoplasm to the ER. In this study, we report that the soluble NS3 transiently associates with the ER to process a native truncated viral polyprotein when NS2B is provided in trans. 

## 2. Materials and Methods 

### 2.1. Cell Culture and Virus

Cell propagation and virus infection experiments were conducted according to the previously described protocol [37]. Briefly, human embryonic kidney 293T (HEK293T ATCC^®^ CRL-3216™) cells (passages 6–15) were cultivated in high-glucose Dulbecco’s modified Eagle medium (DMEM) (Cat#D5546, Millipore Sigma, Burlington, MA, USA) supplemented with 10% heat-inactivated fetal bovine serum (FBS) (Cat#MT35015CV, ThermoFisher Scientific, Waltham, MA, USA) and 1% penicillin/streptomycin (Cat#P0781, Millipore Sigma, Burlington, MA, USA). A stock of lineage I WNV strain NY99 (WNV_NY99_), originally isolated from a crow in New York and propagated in Vero cells [38], was used for all infection experiments. HEK293T cells were infected with WNV_NY99_ at a multiplicity of infection (MOI) of 1 and incubated at 37 °C with 5% CO_2_ and 95% humidity. The resulting supernatants from infected cells were harvested for RNA extraction. The infected cells were either fixed for immunofluorescence assay or lysed for Western blot assays.

### 2.2. Plasmid Construction

Standard molecular biology techniques were used to clone the WNV nonstructural genes [39,40]. Viral RNA was extracted from the supernatant of WNV-infected Vero cells using the QIAamp Viral RNA mini kit (Cat#52904, Qiagen, Hilden, Germany). Extracted RNA was used as a template to generate cDNA with the SuperScript IV first-strand synthesis kit (Cat#18091050, ThermoFisher Scientific, Waltham, MA, USA). The cDNA was used as a template for polymerase chain reaction (PCR) amplification of WNV NS genes. The forward and reverse primers for each WNV gene listed in Table 1 were designed from the WNV reference genome NCBI accession number DQ211652. All forward primers include an optimal translation initiation Kozak sequence and an ATG start codon. The reverse primers were designed to have C-terminal in-frame fusion with the V5/His or GFP tags. Following the amplification of the WNV genes using AmpliTaq Gold^TM^ DNA Polymerase (Cat#N8080259, ThermoFisher Scientific, Waltham, MA, USA), PCR products were cloned into either the pcDNA^TM^3.1/V5-His-TOPO (Cat#K480001, ThermoFisher Scientific, Waltham, MA, USA) or the pcDNA^TM^3.1/CT-GFP-TOPO (Cat#K482001, ThermoFisher Scientific, Waltham, MA, USA) vectors containing the CMV promoter for mammalian expression, according to the manufacturer’s protocol. Plasmid DNA was prepared from large-scale *E. coli* cultures and purified using a plasmid Maxi Kit (Cat#12163, Qiagen). Proper gene orientation and nucleotide sequences of all WNV plasmids were confirmed at the Advanced Studies in Genomics, Proteomics and Bioinformatics (ASGPB) sequencing facility at the University of Hawaii at Manoa. The pcDNA^TM^3-IKKε-FLAG plasmid was constructed as previously described [41] and gifted by Tom Maniatis (Cat#26201, Addgene, Watertown, MA, USA). The host marker plasmids for the ER, Golgi and microtubules (sec61β-RFP, Arf1-RFP and tubulin-GFP) were generously provided by Dr. Nihal Altan-Bonnett, National Institutes of Health, Bethesda, Maryland.

### 2.3. Transient Transfections and Protein Expression

PolyFect transfection reagent (Cat#301105, Qiagen, Hilden, Germany) was used to conduct transient transfections in 24-well plates or 6-well plates with 0.5 to 1.0 μg of plasmid DNA per 2.5 × 10^5^ cells according to the manufacturer’s protocol. Briefly, the plasmid DNA was mixed with the PolyFect reagent in DMEM without FBS for 10 min, diluted with an appropriate amount of growth medium, and the resulting DNA–PolyFect complexes were added to the seeded HEK293T cells grown to 90% confluency. HEK293T cells were either transfected singly or in combinations of two or three recombinant plasmids. The plasmid DNA (sec61β-RFP, Arf1-RFP or tubulin-GFP) was also transfected into infected cells 1 h after infection as described above. Twenty-four hours after transfection, the cells were either fixed with 3.7% paraformaldehyde (PFA) for immunofluorescence (IF) labeling or harvested for Western blot (WB) assays.

### 2.4. Antibodies

Rabbit anti-WNV NS2B (Cat#GTX132060, GeneTex, Irvine, CA, USA) and rabbit anti-WNV NS3 (Cat#GTX131955, GeneTex, Irvine, CA, USA) antibodies were used to detect viral proteins in infected cells. Antibodies against cellular markers used in this study include rabbit anti-calnexin antibody (Cat#C4731, Millipore Sigma, Burlington, MA, USA) mouse anti-PDI antibody (Cat# MA3-019, ThermoFisher Scientific, Waltham, MA, USA), rabbit anti-giantin antibody (Cat#ab80864, Abcam, Cambridge, MA, USA), mouse anti-GM130 antibody (Cat#610822, BD Biosciences, San Jose, CA, USA), rabbit anti-tubulin β antibody (Cat#RB9249, ThermoFisher Scientific, Waltham, MA, USA), mouse anti-tubulin β antibody (Cat#sc-5274, Santa Cruz Biotechnology, Dallas, TX, USA), rabbit anti-IKKε antibody (Cat#ab7891, Abcam, Cambridge, MA, USA) and mouse β-actin antibody (Cat#A5316, Millipore Sigma, Burlington, MA, USA). The primary antibodies against the fused tags are rabbit anti-GFP (Cat#G10362, ThermoFisher Scientific, Waltham, MA, USA), and mouse anti-V5 (Cat#R960-25, ThermoFisher Scientific, Waltham, MA, USA) antibodies. The secondary antibodies are goat anti-rabbit IgG Alexa Fluor 488 (Cat#A11008, ThermoFisher Scientific, Waltham, MA, USA), goat anti-rabbit IgG Alexa Fluor 555 (Cat#A21428, ThermoFisher Scientific, Waltham, MA, USA), goat anti-mouse IgG Alexa Fluor 555 (Cat#A21422, ThermoFisher Scientific, Waltham, MA, USA) and goat anti-rabbit Pacific Blue (Cat# P10994, ThermoFisher Scientific, Waltham, MA, USA) antibodies. The dilutions, vendors and catalog numbers of the primary and secondary antibodies used for IF staining and WB analysis are listed in Table 2.

### 2.5. Indirect Immunofluorescence Assay and Image Acquisition

To examine the subcellular localization of NS3 and NS2B in infected or transfected cells, HEK293T cells were fixed with 3.7% PFA in 1X PBS and incubated with the appropriate primary and secondary antibodies (Table 2). Fixed cells were incubated with primary antibody in 0.1% saponin (Cat#S4521, Millipore Sigma, Burlington, MA, USA), 2% bovine serum albumin (BSA) (Cat# 10735078001) and 1X PBS for 1 h at room temperature followed by fluorophore-tagged secondary antibody in 2% BSA and 1X PBS for 45 min at room temperature. The cells were mounted on slides using Vectashield Mounting Medium with DAPI (Cat#H1200, Vector Laboratories, Burlingame, CA, USA). Slides were viewed and captured using Olympus FV-1000 confocal laser scanning microscope. The slides were viewed, and images were captured with 40× objective and the co-localized cells were confirmed with a 63× objective. To calculate the infection–transfection efficiency (ITE), transfection efficiency (TE), or co-transfection efficiency (Co-TE), the number of cells that were both infected and transfected, singly transfected, or co-transfected, respectively, were counted and converted into a percentage of the total number of DAPI-stained cells. Five to 10 microscopic fields, each containing 15 to 50 cells, were counted. The images were processed and merged with Adobe Photoshop CS3 software according to the policy formulated by the Digital Image Processing and Ethics Group of the Microscopy Society of America (MSA) Education Committee.

### 2.6. Quantitation of Colocalization

Colocalization analysis of the immunofluorescent signals was conducted using FIJI version 2.1.0/1.53c [42], a distribution of ImageJ (National Institutes of Health, Bethesda, USA), using the Coloc 2 plug-in within FIJI. Regions of interest were manually drawn around distinct cells to contain the cytoplasm but exclude the nucleus. To measure the degree of colocalization between pairs of viral proteins and host markers, the PCC using the Costes’ automatic threshold [43] was calculated from 5–10 representative cells. PCC is a well-established measure of correlation that characterizes the degree of overlap between two channels in a microscopy image. PCC ranges from +1 (perfect positive correlation) to −1 (perfect negative correlation), with values close to 0 indicating an absence of correlation. Specifically, PCC values ranging from 0.5 to 1 denote high levels of colocalization, 0.3 to 0.5 denote moderate levels of correlation, and values less than 0.3 denote low or negligible correlation. 

### 2.7. Cell Lysis 

Transfected cells were trypsinized and washed with ice-cold 1X PBS in pre-cooled microcentrifuge tubes and lysed on ice using Mammalian Protein Extraction Reagent (M-PER^TM^, Cat#78503, ThermoFisher Scientific, Waltham, MA, USA) containing 1% proteinase inhibitor cocktail (Cat#4693159001, Millipore Sigma, Burlington, MA, USA) for 1 h on ice with gentle shaking. The microcentrifuge tube containing the lysate was centrifuged at 15,000 rpm at 4 °C for 35 min to pellet the cellular debris. The supernatant was transferred into a chilled microcentrifuge tube and the pellet was discarded. The protein concentration was determined using a Quick Start^TM^ Bradford Protein Assay kit (Cat#5000201, Bio-Rad, Hercules, CA, USA), and bovine serum albumin (BSA) was used as the protein standard. The lysates were then frozen at −80 °C for protein analysis using SDS-PAGE.

### 2.8. Subcellular Fractionation

Infected or transfected HEK293T cells were fractionated as described previously [44], with modification. Briefly, cells of one confluent 100 mm plate were combined by scraping into 500 µL of ice-cold hypotonic buffer containing 1% proteinase inhibitor cocktail (Cat#4693159001, Millipore Sigma, Burlington, MA, USA). The cells were homogenized by 20 passages through a 25-gauge needle (Cat#BD305127, BD Biosciences, San Jose, CA, USA) and the post-nuclear supernatant (PNS) was prepared by centrifugation for 5 min at 1000 g at 4 °C. Aliquots of ≈500 µL of PNS were layered on a discontinuous sucrose density gradient (0.2, 0.4, 0.6, 1.0, 1.4, 1.8 and 2.0 M, 275 µL each) and centrifuged for 2 h at 4 °C at 55,083 g Beckman SW55 rotor. Eleven fractions, ≈220 µL each, were collected from the top. Twenty microliters of each fraction was separated by SDS-PAGE and analyzed by Western blotting.

### 2.9. Western Blot

Approximately 10–20 μg of total protein was mixed with 1X NuPAGE LDS sample buffer (Cat#NP0007, ThermoFisher Scientific, Waltham, MA, USA) and electrophoresed on 4%–12% pre-cast NuPAGE gels (Cat#NP0322BOX, ThermoFisher Scientific, Waltham, MA, USA). Following SDS-PAGE, the proteins were transferred onto a nitrocellulose membrane (Cat#LC2006, ThermoFisher Scientific, Waltham, MA, USA) using the Trans-Blot Turbo Transfer System (Bio-Rad, Hercules, CA, USA), according to the manufacturer’s protocol. The membrane was blocked with Li-Cor Blocking Buffer (Cat#927-60001, Li-Cor Biosciences, Lincoln, NE, USA) for 1 h at room temperature and incubated with primary antibodies (Table 2), followed by incubation with goat anti-rabbit IgG IRDye 800CW (Cat#926-32211) or goat anti-mouse IgG IRDye 680RD (Cat# 926-68170) secondary antibodies (Li-Cor Biosciences, Lincoln, NE, USA) for 1 h at room temperature. The membranes were scanned using the Odyssey CLx Imaging system (Li-Cor Biosciences, Lincoln, NE, USA).

## 3. Results

### 3.1. NS3 and NS2B Localize to the ER in WNV-Infected Cells

**Figure 1:**
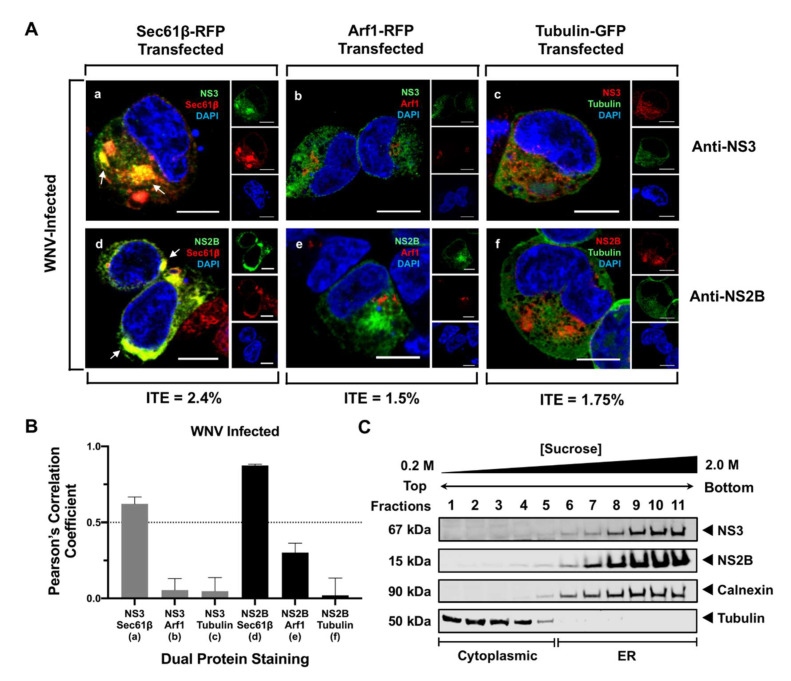
West Nile virus (WNV) nonstructural protein 3 (NS3) and NS2B associate with the ER during infection. (**A**) HEK293T cells were infected with WNV_NY99_ at a multiplicity of infection (MOI) of 1 and after 1 h, transfected with Sec61β-RFP, ER marker, (red; **a**,**d**), Arf1-RFP, a Golgi marker, (red; **b**,**e**), or tubulin-GFP, a cytoskeletal marker, (green; **c**,**f**). Cells were fixed at 24 h after infection and immunostained with an anti-WNV NS3 antibody (green; **a**,**b**, red; **c**) or with an anti-WNV NS2B antibody (green; **d**,**e**, red; **f**). Nuclear DNA was stained with 4,6-diamidino-2-phenylindole (DAPI). Slides were analyzed by confocal laser scanning microscopy. Confocal microscopy images were of optical slice thickness ~1 μm. Scale bar, 10 μm. The main panels represent merged images from three separate channels that are individually shown on the side panels. White arrows indicate colocalization between the green and red channels. The infection–transfection efficiency (ITE) indicating the percentage of cells that were both WNV-infected and transfected for each treatment is listed at the bottom of the panel. Images are representative of two independent infection experiments. (**B**) Pearson’s correlation coefficient (PCC) analysis of the colocalization between NS3 (gray bars) or NS2B (black bars) with cellular markers in WNV infected cells conducted using Coloc 2 (FIJI). PCC values greater than 0.5 (dotted line) indicate a high level of colocalization. Letters in parenthesis beneath each pair of markers correspond to its representative confocal image in Figure 1A. Error bars indicate mean ± standard error of the mean (SEM); n = 5–10 cells per group. (**C**) Cells were infected with WNV_NY99_ at a MOI of 1 and harvested after 48 h. Cell fractions were separated using sucrose gradient ultracentrifugation and subjected to SDS-PAGE before immunoblotting. The viral proteins were detected using anti-WNV NS3 and NS2B antibodies. The cytoplasmic and endoplasmic reticulum (ER) fractions were observed using antibodies against tubulin and calnexin, respectively.

We initially characterized the intracellular localization of NS3 in infected cells by examining its subcellular localization with cellular markers specific for different organelles using high-resolution confocal immunofluorescence (IF) microscopy (Figure 1A). HEK293T cells were infected with WNV_NY99_ at MOI 1 for 1 h and subsequently transfected with plasmids expressing host markers conjugated to RFP or GFP. Sec61β-RFP, Arf1-RFP and tubulin-GFP-expressing plasmids were used to detect the ER, the Golgi apparatus, and cytoplasmic microtubules, respectively (Figure 1A). Twenty-four hours after infection, cells stained for WNV NS3 showed that NS3 predominantly colocalized with the ER marker Sec61β (Figure 1A(a)), but did not associate with the Golgi (Figure 1A(b)) or cytoplasmic markers (Figure 1A(c)). To confirm WNV NS3 localization in the ER and at the virus replication site where other NS proteins are located, an antibody against WNV NS2B was used. IF analyses confirmed that WNV NS2B, a transmembrane protein found within the virus replication complex, colocalized only with Sec61β at the ER, as expected [8] (Figure 1A(d)), and did not colocalize with the Golgi (Figure 1A(e)), or microtubule markers (Figure 1A(f)). We also quantitatively assessed the colocalization between the viral proteins and each host marker in infected cells by calculating the PCC. Consistent with our visual observations, only the PCC for NS3 and NS2B with Sec61β were high, with mean values of 0.62 ± 0.05 and 0.87 ± 0.01, respectively (Figure 1B). To further confirm the association of NS2B and NS3 with the ER during infection, cells infected with WNV_NY99_ for 48 h were subjected to subcellular fractionation and sucrose density gradient ultracentrifugation (Figure 1C). Western blot analysis of solubilized fractions indicated a clear separation between cytoplasmic (fractions 1–5) and ER fractions (fractions 6–11) using antibodies against tubulin and the ER marker, calnexin, respectively (Figure 1C). In these WNV-infected cells, NS2B and NS3 were not observed in the cytoplasmic fractions and were both highly enriched in the ER fractions, corresponding to fractions 6–11 (Figure 1C). Taken together, the IF and WB data confirm previously published data and indicate that NS2B and NS3 are both localized at the ER where virus replication occurs.

### 3.2. WNV NS3 Is Distributed throughout the Cytoplasm, While NS2B Colocalizes with the ER in Transfected Cells

**Figure 2:**
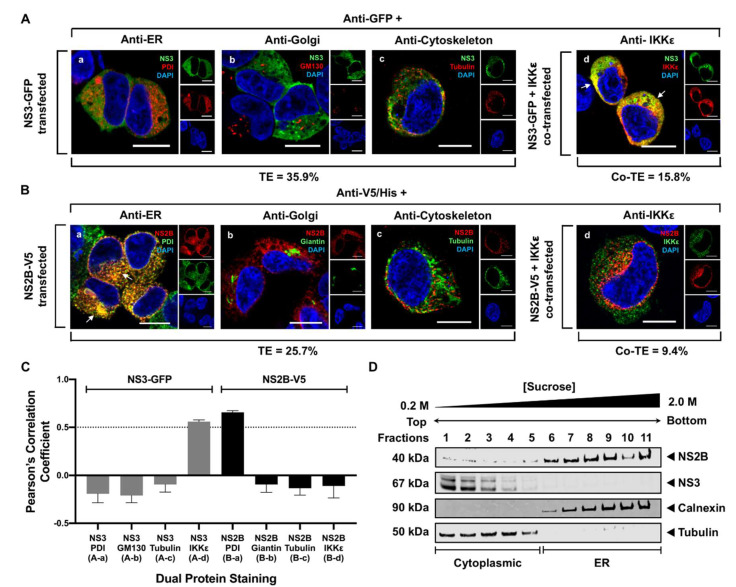
WNV NS3 is distributed throughout the cytoplasm, while NS2B remains in the ER in transfected cells. (**A**) NS3-GFP transfected (**a**–**c**) or NS3-GFP and IKKε-FLAG co-transfected (**d**), HEK293T cells were fixed at 24 h post-transfection and immunostained with antibodies against protein disulfide isomerase (PDI), an ER marker, (red; **a**), anti-GM130, a Golgi apparatus marker, (red; **b**), anti-tubulin, a cytoskeletal marker, (red; **c**), or anti-IKKε, a soluble cytoplasmic protein marker, (red; **d**). (**B**) NS2B-V5/His transfected (**a**–**c**) or NS2B-V5/His and IKKε-FLAG co-transfected (**d**) HEK293T cells were fixed 24 h after transfection and immunolabeled with rabbit anti-PDI (green; **a**), anti-giantin (green; **b**), anti-tubulin (green; **c**) or anti-IKKε (green; **d**) antibodies to visualize the ER, Golgi apparatus, cytoskeleton or the cytoplasmic protein kinase IKKε protein, respectively. Visualized viral and host proteins are listed on the top right corner of each panel. Nuclear DNA was labeled with DAPI. Confocal microscopy images were of optical slice thickness ~1 μm. Scale bar, 10 μm. The main image depicts the merged image from three channels separately shown on the side. White arrows indicate colocalization between the green and red channels. The transfection efficiency (TE) for cells transfected with one construct or the co-transfection efficiency (Co-TE) for cells co-expressing the viral protein and IKKε are listed at the bottom of each panel. Images are representative of two independent transfection experiments. (**C**) Colocalization analysis between viral proteins and host markers was conducted using Coloc 2 (FIJI). Pearson’s correlation coefficient (PCC) was calculated for each indicated pair of proteins in NS3-GFP (gray bars) or NS2B-V5 (black bars) transfected cells. PCC values larger than 0.5 (dotted line) indicate a high probability that pixels from the red and green channels overlap. Parenthesized letters link each PCC value to its corresponding confocal image in Figure 2A or Figure 2B. Error bars indicate mean ± SEM; n = 5–10 cells per group. (**D**) NS2B-GFP or NS3-V5 singly transfected HEK293T cells were lysed 48 h post-transfection and fractionated by sucrose gradient centrifugation. Cell fractions were analyzed by Western blot using mouse anti-GFP or rabbit anti-V5 antibodies to detect the NS2B and NS3 proteins, respectively. The cytoplasmic and ER fractions were visualized using anti-tubulin and anti-calnexin antibodies, respectively.

To examine the subcellular localization of NS3 or NS2B in HEK293T cells, we independently expressed NS3 or NS2B fused to a GFP or V5/His epitope, respectively (Figure 2A,B). Twenty-four hours post-transfection, fixed cells were labeled with antibodies against cellular markers for the ER [protein disulfide isomerase (PDI)], the Golgi apparatus [Golgi matrix 130 (GM130) or giantin] and cytoskeleton (tubulin) and visually analyzed using confocal microscopy. Quantitative analysis of the colocalization between NS3-GFP or NS2B-V5 with these cellular markers was also conducted by measuring the PCC for each pair of proteins (Figure 2C). In NS3-transfected cells, NS3 was in the cytoplasm but not at the ER or Golgi (Figure 2A(a–c)). To verify that NS3 did not associate with the cytoskeleton in the cytoplasm, NS3 and IKKε (a known soluble host protein) were co-expressed in HEK293T cells. At 24 h, NS3 colocalized with the IKKε protein (PCC = 0.56 ± 0.02), indicating that NS3 is a soluble protein that is diffusely distributed in the cytoplasm (Figure 2A(d)). In contrast, NS2B, a membrane-bound protein, was observed to colocalize specifically with the ER marker PDI (PCC = 0.66 ± 0.02), confirming the ER localization of NS2B even when it was individually expressed (Figure 2B(a)). There was no apparent colocalization of NS2B with the Golgi (Figure 2B(b)), the cytoskeleton (Figure 2B(c)), or with IKKε in co-expressing cells (Figure 2B(d)), which was verified by their slightly negative correlation coefficients (Figure 2C). The ER localization of NS2B and the cytoplasmic distribution of NS3 were also confirmed using subcellular fractionation of NS2B- and NS3-transfected cells (Figure 2D). Western blot analysis of the sucrose density gradient fractions was performed using extracted soluble tubulin as a cytoplasmic marker (fractions 1–5) and using calnexin as the ER marker (fractions 6–11) (Figure 2D). As expected, NS2B was detected in the calnexin-enriched fractions 6–11. On the other hand, NS3 and tubulin were both visible in fractions 1–4 (Figure 2D), indicating that NS3 is in the cytoplasm when expressed in the absence of other viral proteins.

### 3.3. NS3 Localizes to the ER When NS2B Is Provided in Trans and in Cis

To confirm that NS4A and NS4B do not play a role in recruiting NS3 to the ER, cells were transfected with NS3, NS4A or NS4B fused to a V5/His epitope and the ER marker plasmid, Sec61β-RFP. Colocalization of NS3 with the ER was analyzed both visually and quantitatively using confocal IF microscopy and PCC measurements, respectively. In the presence of the ER-associated NS4A or NS4B proteins, NS3 remained diffusely distributed throughout the cytoplasm and did not localize at the ER (Figure S1A,C). Consistently, quantitation of colocalization between NS3 and Sec61β by PCC showed negative correlation coefficients (Figure S1B,D). Collectively, these results demonstrate that neither NS4A nor NS4B are involved in the recruitment of NS3 to the ER.

**Figure 3:**
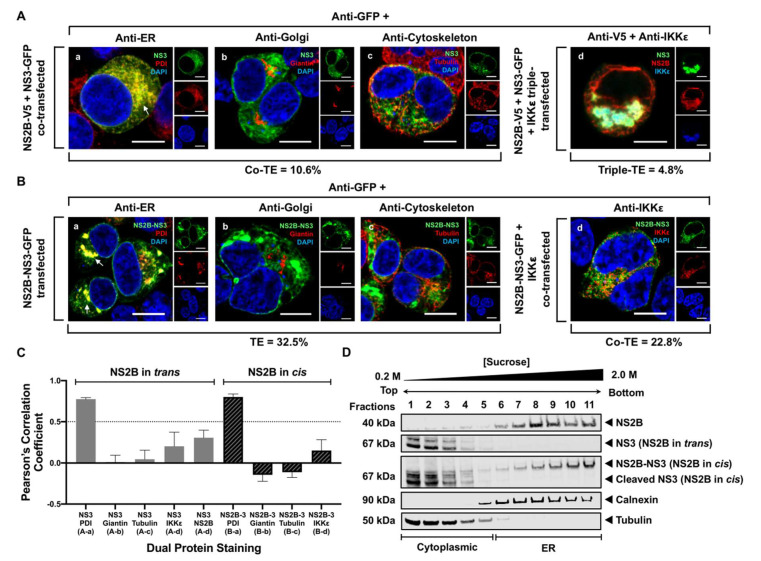
NS3 localizes to the ER when NS2B is provided in trans and in cis. (**A**) HEK293T cells were co-transfected with NS3-GFP and NS2B-V5 expressing plasmids (**a**–**c**). At 24 h post-transfection, cells were fixed and co-stained with anti-GFP and antibodies against PDI (red; **a**), giantin (red; **b**) or tubulin (red; **c**). HEK293T cells were also triple-transfected with NS3-GFP, NS2B-V5/His and IKKε-FLAG expressing plasmids (**d**) and triple stained with anti-GFP (green; **d**), anti-V5/His (red; **d**) and anti-IKKε (blue; **d**) antibodies. (**B**) HEK293T cells were transfected with NS2B-NS3-GFP (**a**–**c**) or co-transfected with NS2B-NS3-GFP and IKKε-FLAG expressing plasmids (**d**) and processed for immunofluorescence at 24 h after transfection using antibodies against PDI (red; **a**), giantin (red; **b**), tubulin (red; **c**) and IKKε (red; **d**). The detected viral and host proteins are listed on the top right corner of each panel. White arrows indicate colocalization between the GFP-tagged viral protein and the ER marker. Nuclear DNA was labeled with DAPI. Scale bar represents 10 μm. The main panels depict merged images with the side panels showing the individual channels. The transfection efficiency (TE), the co-transfection efficiency (Co-TE) and the triple-transfection efficiency (Triple-TE) for cells expressing one, two, or three gene constructs, respectively, are listed at the bottom of the panel. Images are representative of two independent transfection experiments. (**C**) Quantitation of colocalization between NS3 and cellular markers when NS2B is provided in trans (gray bars) or in cis (diagonally striped bars) was determined using the Pearson’s correlation coefficient (PCC). Letters in parenthesis underneath each pair of proteins indicate its corresponding confocal image in Figure 3A or Figure 3B. PCC values greater than 0.5 (dotted line) indicate a high level of colocalization. Error bars indicate mean ± SEM; n = 5–10 cells per group. (**D**) HEK293T cells expressing both NS3-V5 and NS2B-GFP with NS2B provided in trans or cells expressing NS2B-NS3-GFP with NS2B provided in cis were lysed 48 h post-transfection and subjected to sucrose density gradient ultracentrifugation. Aliquots of fractions collected from the top of the gradient were analyzed by Western blotting. Rabbit anti-tubulin and anti-calnexin antibodies were used to distinguish between the cytoplasmic and ER fractions, respectively, and antibodies against the fused tag (GFP or V5/His) were used to detect the viral protein.

To examine the potential role of NS2B in recruiting NS3 from the cytoplasm to the ER, we co-expressed NS3 with NS2B fused to a GFP or V5/His epitope, respectively. NS2B was provided in trans (Figure 3A) and cis (Figure 3B). Colocalization of NS3 with specific cellular markers for the ER (PDI), Golgi (giantin), and cytoskeleton (tubulin) was visually analyzed by confocal IF microscopy and quantitatively verified by calculating the PCC in these co-transfected cells 24 h after transfection. When NS2B was provided in trans, NS3 no longer showed diffused cytoplasmic distribution and localized exclusively with the ER (Figure 3A(a)). The mean PCC for NS3 and PDI was 0.78 ± 0.02, confirming the colocalization of NS3 with the ER in these co-transfected cells (Figure 3C). NS3 was neither observed at the Golgi apparatus nor in the cytoplasmic cytoskeleton (Figure 3A(b,c)), and showed low levels of colocalization (Figure 3C). To confirm that NS3 was no longer predominantly in the cytoplasm, cells were transfected with NS2B, NS3 and the cytoplasmic IKKε expressing plasmids (Figure 3A(d)). Triple-channel confocal imaging of transfected HEK293T cells detecting NS2B, NS3, and IKKε demonstrated higher overlap between the green (NS3) and red (NS2B) fluorophores, compared to NS3 and the blue fluorescent IKKε (Figure 3A(d) and Figure S2). This was supported by quantitative analysis in which NS3 showed a slightly higher level of colocalization with NS2B than with IKKε, even though the positive PCC values in the triple transfection are much lower compared to those in the dual transfection (Figure 3C). This disparity may be due to the presence of IKKε in the triple transfection. NS3 and IKKε previously showed a corresponding PCC value of 0.56 (Figure 2C), clearly indicating that there may be some degree of interaction between NS3 and IKKε that may contribute to the dampened PCC value observed for NS3 and NS2B in the triple-transfected cells. When NS2B was provided in cis, NS3 was mostly at the ER (Figure 3B(a)), as observed previously (Figure 3A(a)). Similarly, NS3 was not localized to the Golgi (Figure 3B(b)), cytoskeleton (Figure 3B(c)), or in the cytoplasm (Figure 3B(d)). These observations were confirmed as NS3 exhibited high levels of colocalization only with the ER marker (PCC = 0.80 ± 0.04) and displayed low or negative colocalization with the other cellular markers when NS2B was provided in cis (Figure 3C). Subcellular fractionation and sucrose density gradient ultracentrifugation assay was also conducted on lysates from co-expressing cells when NS2B was provided in trans and in cis. Western blot analysis of the subcellular fractions revealed that NS3 was observed in cytoplasmic fractions 1-5 and did not appear to be in the ER fractions when NS2B was provided in trans, while NS2B was detected in the calnexin-enriched ER fractions 6–11 as expected (Figure 3D). Interestingly, when NS2B was in cis, NS3 was detected in the ER fractions 6–11, and a smaller band at approximately 67 kDa also appeared in cytoplasmic fractions 1–5 (Figure 3D), potentially corresponding to the cleaved NS3 product. These biochemical data indicate that the NS3 is released to the cytoplasm once it is auto-cleaved from the viral NS2B-NS3 polyprotein precursor. Based on the IF and biochemical observations, NS3 appears to be peripherally recruited by NS2B to the ER to perform its key enzymatic functions essential for virus replication. 

**Figure 4:**
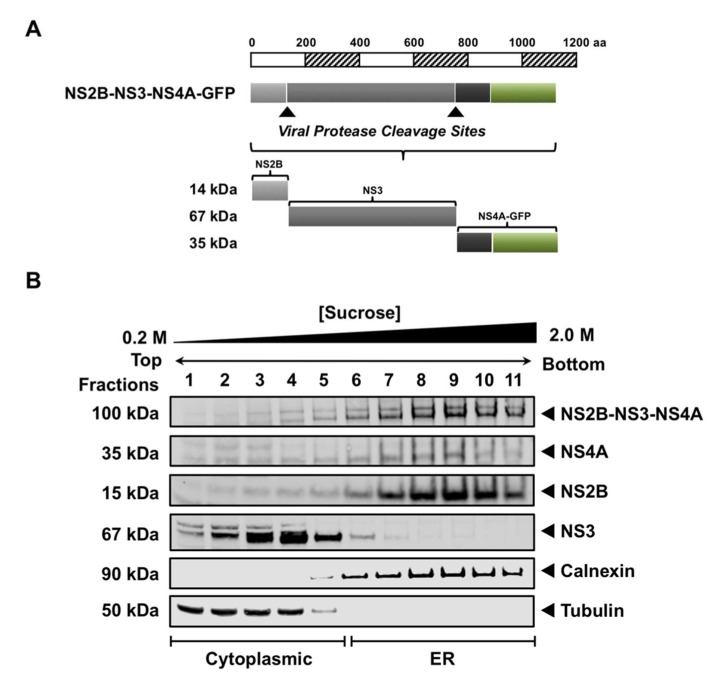
Processing of NS3 from the viral polyprotein precursor does not contribute to its ER localization. (**A**) Schematic representation of the GFP-tagged NS2B-NS3-NS4A polyprotein. The GFP epitope is indicated by the green box. Construct is drawn to scale according to the number of amino acid residues. The viral protease cleavage sites at the NS2B-NS3 and NS3-NS4A junctions are marked by the black arrowheads. (**B**) NS2B-NS3-NS4A-GFP transfected HEK293T cells were lysed 48 h post-transfection and subjected to sucrose density gradient ultracentrifugation. Aliquots of fractions collected from the top of the gradient were analyzed by Western blotting. The cytoplasmic and ER fractions were distinguished using anti-tubulin and anti-calnexin antibodies, respectively. The unprocessed full-length NS2B-NS3-NS4A polyprotein and processed NS4A protein were detected using an anti-GFP antibody. Processed NS2B and NS3 proteins were visualized using anti-WNV NS2B and NS3 antibodies.

To evaluate whether the sequential processing of NS2B-NS3-NS4A is involved in the ER localization of NS3, we constructed a GFP-tagged NS2B-NS3-NS4A plasmid (Figure 4A) and expressed it in HEK293T cells for 48 h. Characterization of the subcellular fractions using WB analysis revealed that the full-length polyprotein was partially processed to release NS2B, NS3, and NS4A proteins, corresponding to 14, 67, and 35 kDa bands, respectively (Figure 4B). NS2B and NS4A, both membrane-bound proteins, were observed in the ER fractions 6–11 (Figure 4B), as expected. Interestingly, NS3 was detected in the cytoplasmic fractions 1–5 (Figure 4B), indicating that sequential processing of NS3 from the NS2B-NS3-NS4A polyprotein precursor, a form that mimics the native processing of NS3, was not involved in its ER localization.

### 3.4. NS3 Is Proteolytically Active When NS2B Provided in Trans

**Figure 5:**
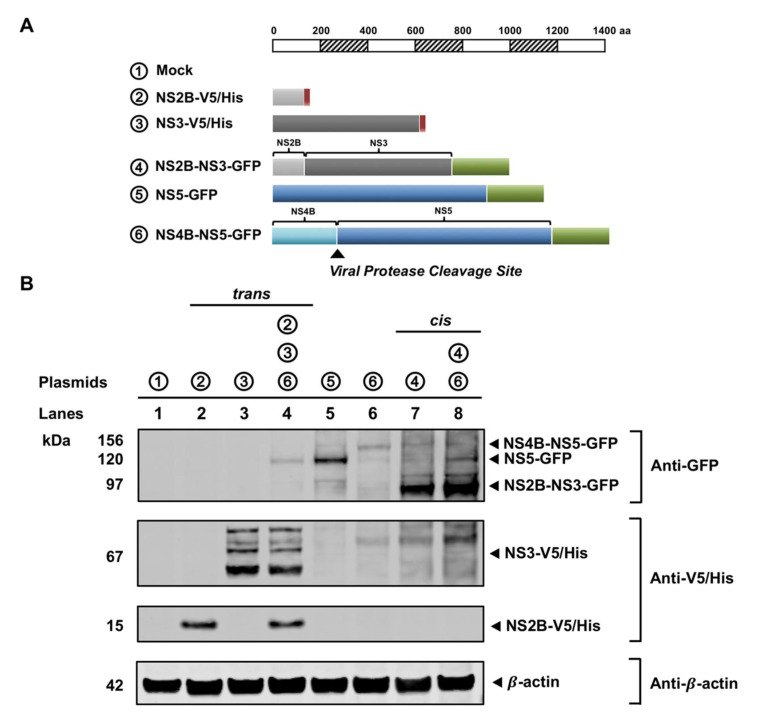
NS3 is a functional viral protease when NS2B is provided in trans. (**A**) Schematic diagram depicting the V5/His or GFP-tagged WNV NS constructs used in this proteolytic processing assay. Constructs are drawn to scale according to the number of amino acid residues. The V5 or GFP tags are indicated by red or green boxes, respectively. The viral protease cleavage site between NS4B and NS5 is marked by the black arrowhead. (**B**) HEK293T cells were transfected with various combinations of WNV NS constructs (listed at the top of the panel) and cell lysates were harvested 48 h post-transfection. Cleavage of NS4B-NS5-GFP by NS3-V5/His when NS2B was provided in trans (lanes 2–4) or in cis (lanes 7–8) was assessed by Western blotting using anti-GFP and anti-V5/His antibodies to detect the transfected viral proteins and cleaved products. β-actin served as an internal loading control. Molecular weights (kDa) are given on the left side of each panel.

To evaluate whether the recruitment of NS3 to the ER by NS2B resulted in a functional viral protease, we constructed an ER-membrane bound, GFP-fused NS4B-NS5 polyprotein containing a viral protease cleavage site (Figure 5A). It is known that NS3 without its NS2B cofactor is proteolytically inactive [22,34,36]. NS3 requires the presence of NS2B to cleave at specific sites within the viral polyprotein precursor, including the junction site between NS4B and NS5 [22,23,35]. The proteolytic capacity of NS3 when NS2B was provided in trans and in cis was examined in cells co-expressing the NS4B-NS5 polyprotein (Figure 5B). WB analysis of the total cell lysates where NS2B was in trans revealed bands at approximately 15 and 67 kDa, representing NS2B (lane 2 and 4) and NS3 proteins (lane 3 and 4), respectively, when stained with an anti-V5/His antibody. When probed with an anti-GFP antibody, a large 156 kDa band (lane 6) corresponding to the full-length GFP-fused NS4B-NS5 polyprotein was not detected, but a smaller 127 kDa band was visible, correlating to the single GFP-fused NS5 protein (lane 4 and 5). To reaffirm the proteolytic function of NS3, WB analysis of the lysate from cells where NS2B was in cis revealed the absence of the larger 156 kDa full length NS4B-NS5 band and the presence of the 127 kDa NS5-GFP band using an antibody against GFP (lane 8). The same processing activity was also observed when NS3 was fused to GFP (Figure S3), indicating that the fused tag did not have any influence on the proteolytic activity of NS3. These results clearly demonstrate that NS3 recruited to the ER is indeed enzymatically active. 

## 4. Discussion

NS3 plays central roles in viral polyprotein processing and viral RNA synthesis at the ER, as it serves as the viral protease when it is associated with its NS2B cofactor and contains RNA helicase activity essential for unwinding the RNA during replication. The predicted membrane topology of NS3 indicates that NS3 is a hydrophilic protein lacking transmembrane domains, implying that NS3 is a soluble protein [45]. However, NS3 may be enwrapped in the replication complex by the specialized membrane compartments at the ER during infection [8], which is supported by this study showing that NS3 localized to the ER in infected cells (Figure 1). NS3 strictly interacts with NS2B for proteolytic activity [22], and with NS5 for RNA synthesis [17,46] but its reported interactions with other membrane-embedded WNV NS proteins are not conclusive [31,32,33]. In this study, NS4A and NS4B did not colocalize with NS3 at the ER (Figure S1), which is consistent with published data [33]. However, in the presence of virus-induced compartments, NS4A and NS4B may assist NS3 in anchoring the replication complex to the ER membrane [31,32,47]. Despite these potential interactions of NS3 and other viral proteins, how NS3 is recruited from the cytoplasm to the ER to execute its essential enzymatic roles has not yet been fully explored. Using HEK293T cell-based transfection and high-resolution confocal microscopy with PCC analysis and biochemical assays, we demonstrate that NS2B may be the key viral protein that recruits NS3 to the ER where virus replication occurs.

Our confocal data revealing the localization of NS3 in the cytoplasm (Figure 2) are consistent with previous studies reporting that flavivirus NS3 exhibits diffuse cytoplasmic staining patterns in infected and NS3-transfected cells [8,33,48]. Another study using Japanese encephalitis virus (JEV), a member of the same serocomplex as WNV, also demonstrated the accumulation of NS3 in the perinuclear region in infected cells [49]. In addition, a previous study was not able to observe any colocalization between singly expressed NS3 and other cellular cytoskeleton proteins, vesicle-associated proteins or lipid raft-related proteins [33], suggesting that NS3, when expressed alone, may be nonspecifically distributed throughout the cytoplasm. However, in our study, we clearly demonstrate that NS3 is a cytoplasmic protein that was not in the cytoskeleton, and it perfectly co-localized with cytoplasmic IKKε (Figure 2). This colocalization indicates that NS3 and IKKε may have some interaction, which is consistent with previously published data [50]. Our data also reveal that NS3 was not in the ER or Golgi apparatus in the absence of other viral proteins (Figure 2).

This study is the first to show the ER localization of WNV NS3 when NS2B is provided in trans (Figure 3), and that the recruited NS3 is enzymatically active (Figure 5). The interaction of NS3 and NS2B occurs via the N-terminal NS3 protease domain with the hydrophilic 40-amino acid segment in NS2B [34], indicating that this interaction may be transient, and these data suggest that this is the case (Figure 3). Studies have also shown that NS3 is released after cleavage from the NS2B-NS3-NS4A polyprotein to be functionally active in the ER [8,23]. However, our study shows that processing of NS3 from the polyprotein was not involved in its ER association (Figure 4), but that NS3 is still enzymatically active (Figure 5). These observations indicate that other factors may be involved in the envelopment of NS3 in the ER. We propose that the released NS3 after cleavage remains in the virus-induced membrane structures once it is enwrapped by it. This suggestion is consistent with the published data showing that NS3 is localized to the specialized virus-induced membrane compartments [8]. This may also explain why we observed NS3 predominantly in the ER fractions during infection (Figure 1).

## 5. Conclusions

Conditions that interrupt the NS2B/NS3 interaction may prevent NS3 from associating with the ER, possibly resulting in the inactivation of NS3 protease and helicase activity, which may lead to the attenuation of viral polyprotein processing and virus replication. The interaction between NS2B and NS3 may be a promising antiviral target. Indeed, a previous study using structure-based screening and cell-based replication assays identified a noncompetitive inhibitor, SK-12, that targeted the NS2B-binding site of NS3, resulting in inhibited viral replication [51]. Information from these studies will not only expand our basic knowledge on the spatial relationship between the nonstructural proteins and the virus-induced membrane compartments at the ER in infected cells, but also aid in the discovery of therapeutics for flaviviruses.

## Figures and Tables

**Table 1:** Sequences of primers used for the construction of WNV nonstructural (NS) gene constructs.

WNV Gene Constructs	Primer	Nucleotide Position *	Primer Sequence (5’ → 3’)
**NS2B**	forward	4219–4234	accaccatg ^a^ GGATGGCCCGCAACTG
	reverse	4686–4611	TCTCTTTGTGTATTGGAGAGTTATCC
**NS3**	forward	4612–4631	accaccatg ^a^ GGAGGCGTGTTGTGGGACAC
	reverse	6444–6468	GACGTTTTCCCGAGGCGAAGTCCTTG
**NS2B-NS3**	forward	4219–4234	accaccatg ^a^ GGATGGCCCGCAACTG
	reverse	6444–6468	GACGTTTTCCCGAGGCGAAGTCCTTG
**NS2B-NS3-NS4A**	forward	4219–4234	accaccatg ^a^ GGATGGCCCGCAACTG
	reverse	6824–6846	GACGTTGCTTCTCTGGCTCAGGAA
**NS4B-NS5**	forward ^b^	6865–6882	accaccatg ^a^ *gct* ^c^ CTAGCCGTGTTCCTGATT
	reverse	10370–10395	GCAGTACTGTGTCCTCAACCAAAGTTG
**NS5**	forward	7681–7698	accaccatg ^a^ GTGGGGCAAAAGGACGC
	reverse	10370–10395	GCAGTACTGTGTCCTCAACCAAAGTTG

^a^ Kozak sequence is lowercase and the viral sequences are in uppercase; ^b^ NS4B forward primer includes the 17-amino acid signal sequence at the C-terminal end of NS4A; ^c^ Codon filling nucleotides are lowercase and italicized. * GenBank Accession No. DQ211652.

**Table 2:** Antibodies used for immunofluorescence (IF) and western blot (WB) staining.

Protein	Primary Antibody	Catalog Number	IF	WB	Secondary Antibody	Catalog Number	IF	WB
			Dilutions				Dilutions	
**WNV NS2B**	rabbit polyclonal	GTX132060 ^a^	1:100		anti-rabbit IgG AF 488	1:1000	A11008 ^b^	
			1:100		anti-rabbit IgG AF 555	A21428 ^b^	1:1000	
				1:3000	anti-rabbit IRDye 800CW	926-32211 ^g^		1:10,000
**WNV NS3**	rabbit polyclonal	GTX131955 ^a^	1:100		anti-rabbit IgG AF 488	A11008 ^b^	1:1000	
			1:100		anti-rabbit IgG AF 555	A21428 ^b^	1:1000	
				1:3000	anti-rabbit IRDye 800CW	926-32211 ^g^		1:10,000
**Calnexin**	rabbit polyclonal	C4731 ^c^	1:100		anti-rabbit IgG AF 488	A11008 ^b^	1:1000	
				1:2000	anti-rabbit IRDye 800CW	926-32211 ^g^		1:10,000
**Protein Disulfide Isomerase (PDI)**	mouse monoclonal	MA3-019 ^b^	1:100		anti-mouse IgG AF 555	A21422 ^b^	1:1000	
**Giantin**	rabbit polyclonal	ab80864 ^d^	1:100		anti-rabbit IgG AF 555	A21428 ^b^	1:1000	
**Golgi matrix 130 (GM130)**	mouse monoclonal	610822 ^e^	1:100		anti-mouse IgG AF 555	A21422 ^b^	1:1000	
**Tubulin β**	rabbit polyclonal	RB9249 ^b^	1:100		anti-rabbit IgG AF 555	A21428 ^b^	1:1000	
	mouse monoclonal	sc-5274 ^f^		1:1000	anti-mouse IRDye 680RD	926-68170 ^g^		1:10,000
**IκB kinase** **subunit ε (IKKε)**	rabbit polyclonal	ab7891 ^d^	1:100		anti-rabbit IgG AF 555	A21428 ^b^	1:1000	
			1:100		anti-rabbit IgG AF 488	A11008 ^b^	1:1000	
			1:100		anti-rabbit IgG Pacific Blue	P10994 ^b^	1:500	
**β-actin**	mouse monoclonal	A5316 ^c^		1:2000	anti-mouse IRDye 680RD	926-68170 ^g^		1:10,000
**Green fluorescent protein (GFP)**	rabbit polyclonal	G10362 ^b^	1:100		anti-rabbit IgG AF 488	A11008 ^b^	1:1000	
				1:4000	anti-rabbit IRDye 800CW	926-32211 ^g^		1:10,000
**V5/His epitope**	mouse monoclonal	R960-25 ^b^	1:100		anti-mouse IgG AF 555	A21422 ^b^	1:1000	
				1:2000	anti-mouse IRDye 680RD	926-68170 ^g^		1:10,000

^a^ Genetex, Irvine, CA, USA; ^b^ ThermoFisher Scientific, Waltham, MA, USA; ^c^ Millipore Sigma, Burlington, MA, USA; ^d^ Abcam, Cambridge, MA, USA; ^e^ BD Biosciences, San Jose, CA, USA; Cambridge, MA, USA; ^f^ Santa Cruz Biotechnology, Dallas, TX, USA; ^g^ Li-Cor Biosciences, Lincoln, NE, USA; IF, immunofluorescence; WB, Western blotting; AF, Alexa Fluor.

## Data Availability

Not applicable.

## Supplementary Materials

The following are available online at https://www.mdpi.com/1999-4915/13/2/216/s1, Figure S1. NS4A or NS4B does not recruit NS3 to the ER. Figure S2. NS3 shows slightly higher colocalization with NS2B than with IKKε. Figure S3. Fused Tag Does Not Influence the Proteolytic Activity of NS3 when NS2B is provided in trans.
