# Supplementary material for: Potential Dual Role of West Nile Virus NS2B in Orchestrating NS3 Enzymatic Activity in Viral Replication
Source: Viruses. 2021 Jan 31;13(2):216. doi: 10.3390/v13020216 (PMC7911885; doi:10.3390/v13020216)
Supplement: Supplementary file 1 [file viruses-13-00216-s001.pdf]

SUPPLEMENTARY MATERIALS

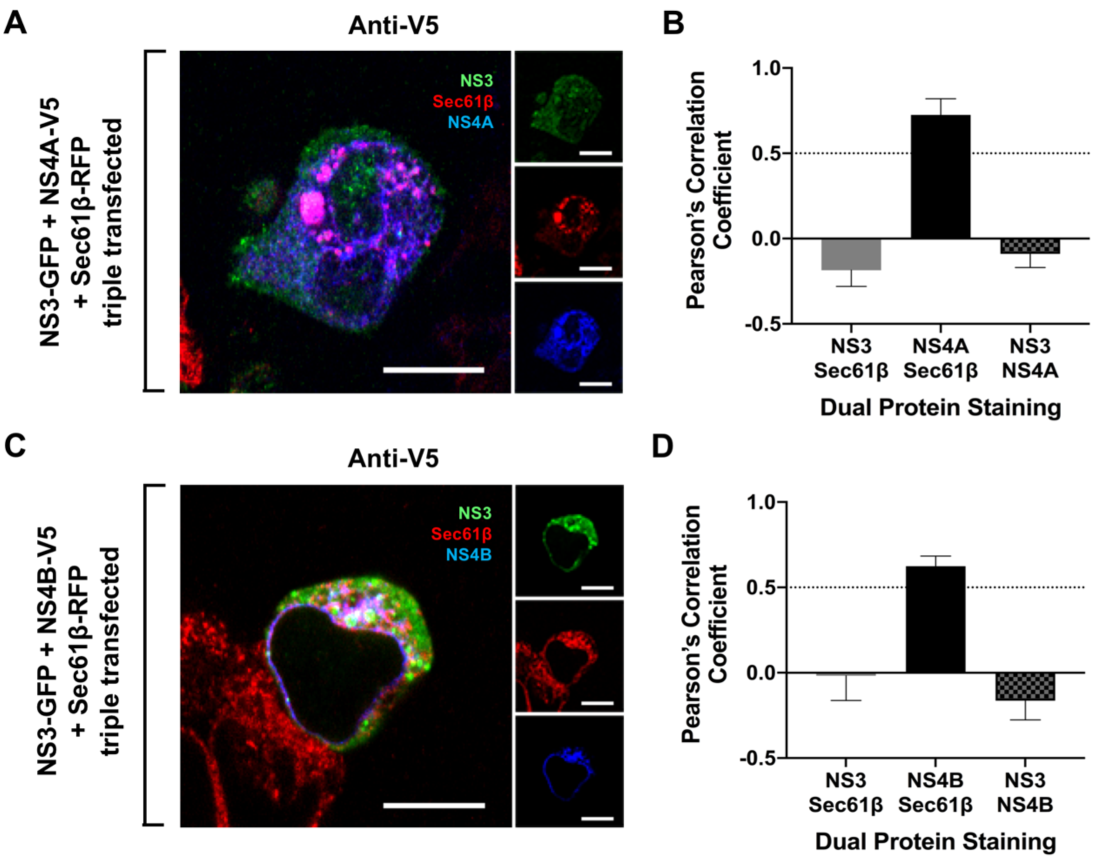

Figure S1. NS4A or NS4B does not recruit NS3 to the ER. (A, C) HEK293T cells were triple-transfected with NS3-GFP, Sec61β-RFP (ER marker) and NS4A-V5 or NS4B-V5 expressing plasmids. At 24 hours post-transfection, cells were fixed and stained with anti-GFP (green) and anti-V5/His (blue) antibodies. Visualized proteins are listed on the top right corner of each panel. Confocal microscopy images were of optical slice thickness ~1 μm. Scale bar, 10 μm. The main image depicts the merged image from three channels that are individually shown on the side panels. (B, D) Pearson's correlation coefficient (PCC) analysis of the colocalization between each indicated pair of proteins was conducted using Coloc 2 (FIJI). PCC values greater than 0.5 (dotted line) indicate a high level of colocalization. Error bars indicate mean ± SEM; n = 3-5 cells per group.

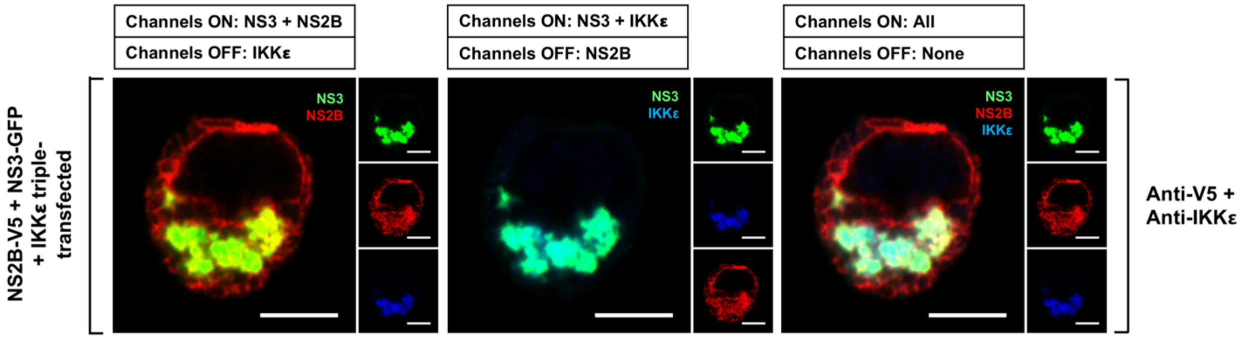

Figure S2. NS3 shows slightly higher colocalization with NS2B than with IKKε. HEK293T cells were triple-transfected with NS3-GFP, NS2B-V5/His and IKKε expressing plasmids and stained with anti-V5/His (red) and anti-IKKε (blue) antibodies. The left main panel highlights only the green and red channels, the middle main panel shows the green and blue channels and the right panel depicts the merged image from all channels. All three panels depict a merged image of the same cell, with the smaller side panels showing each individual channel. Visualized proteins are listed on the top right corner of each panel. Yellow and cyan fluorescence indicates the colocalization between NS3 and NS2B, and NS3 and IKKε, respectively. Scale bar represents 10 µm.

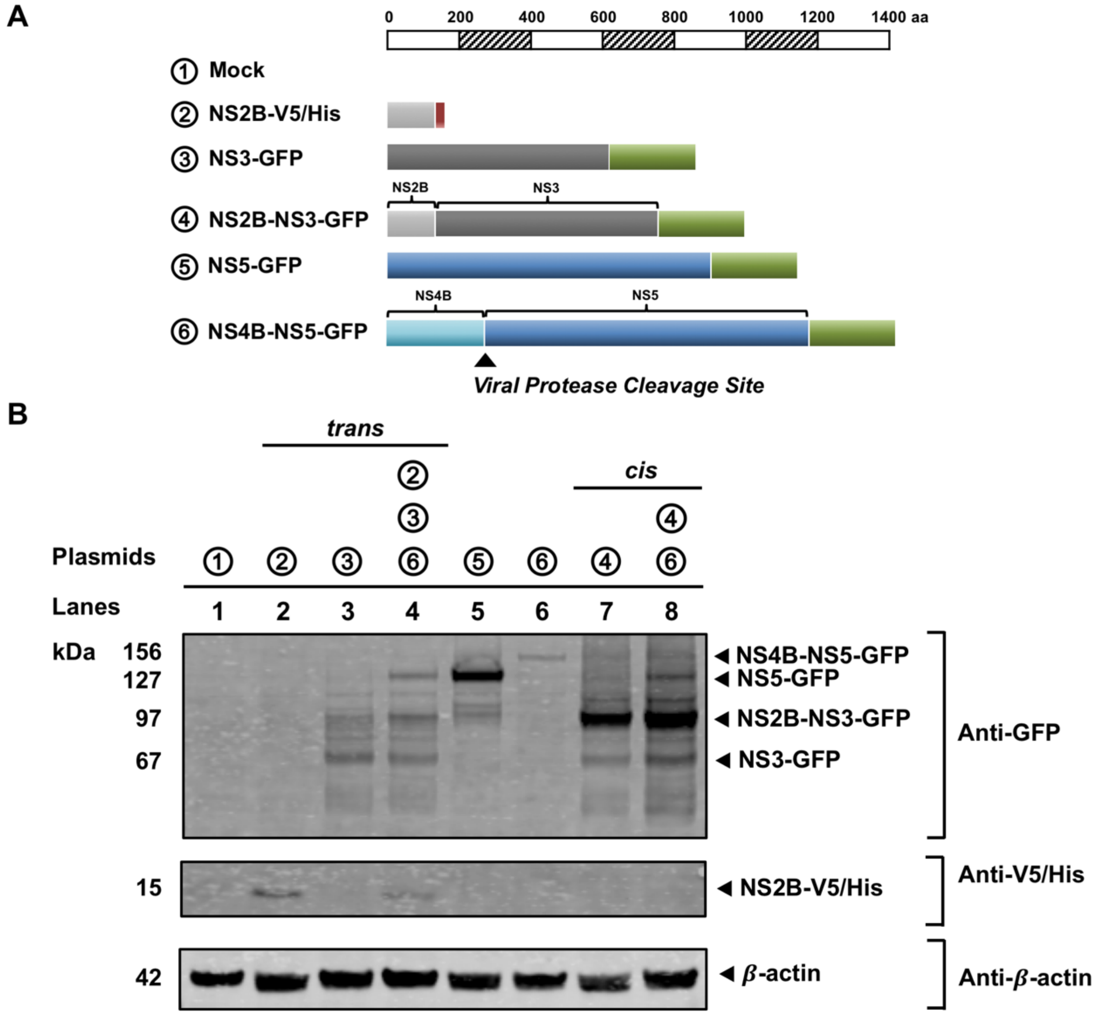

Figure S3. Fused Tag Does Not Influence the Proteolytic Activity of NS3 when NS2B is provided in *trans*. (A) Schematic representation of the V5/His or GFP-tagged WNV NS constructs used in this proteolytic assay. Constructs are drawn to scale according to the number of amino acid residues. The V5 or GFP tags are indicated by red or green boxes, respectively. The viral protease cleavage site between NS4B and NS5 is marked by the black arrowhead. (B) HEK293T cells were transfected with various combinations of WNV NS constructs (listed at the top of the panel) and cell lysates were harvested 48 hours post-transfection. Cleavage of NS4B-NS5-GFP by NS3-GFP when NS2B was provided in *trans* (lanes 2-4) or in *cis* (lanes 7-8) was assessed by western blotting using anti-GFP and anti-V5/His antibodies to detect the transfected viral proteins and cleaved products. Detection of β-actin served as an internal loading control. Molecular weights (kDa) are given on the left side of each panel.
